# Efficacy and Safety of Variable Treatment Options in the Prevention of Hepatic Encephalopathy: A Systematic Review and Network Meta-Analysis

**DOI:** 10.7759/cureus.53341

**Published:** 2024-01-31

**Authors:** Mohamed Hammd, Abdelwahap Elghezewi, Ahmed Abdulhadi, Abdelwahhab Alabid, Abdulfatah Alabid, Yasra Badi, Ibrahem Kamal, Mohamed Hesham Gamal, Khalid Mohamed Fisal, Mohamed Mujtaba, Ahmed Sherif, Wesam Frandah

**Affiliations:** 1 Internal Medicine/Gastroenterology, Marshall University Joan C. Edwards School of Medicine, Huntington, USA; 2 Internal Medicine, Faculty of Medicine, Tripoli University, Tripoli, LBY; 3 Internal Medicine, All Saints University School of Medicine, Dominica, USA; 4 General Medicine, Al-Azhar University, Alexandria, EGY; 5 Pharmacology and Therapeutics, Faculty of Pharmacy, Tanta University, Banha, EGY; 6 Pharmacology and Therapeutics, Faculty of Pharmacy, Deraya University, Minia, EGY

**Keywords:** network meta-analysis, minimal hepatic encephalopathy, ammonia level, liver cirrhosis, hepatic encephalopathy

## Abstract

There are no guidelines for the most effective medication to reduce hepatic encephalopathy (HE) or the associated mortality. The purpose of this study is to determine the most effective possible treatment among the single treatment options or the combined treatment options for decreasing the morbidity and mortality of HE. We evaluated the outcomes by various parameters such as the quality of life, reduction in ammonia, all causes of mortality, adverse events, reversal of minimal HE, and development of overt HE. We systematically searched PubMed, Cochrane, Web of Science, and Scopus till the 19th of January 2023 for studies that assess various treatment options for HE. Data were extracted from eligible studies and pooled in a frequentist network meta-analysis as standardized mean difference (SMD) and their 95% confidence interval (CI) using the MetaInsight web-based tool. The Cochrane Tool was used to assess the randomized controlled trials' quality (RCT), while the NIH tool was used to assess the quality of the included cohort studies. Utilizing the R software, the network meta-analysis was conducted. In addition to a significant variation in cases of (Lactulose and Rifaximin) compared with Rifaximin (RR= 0.39, 95% CI [0.17; 0.89]), the results demonstrated a significantly lower incidence of overt HE in (Lactulose and Rifaximin) compared with placebo (RR=0.19, 95% CI [0.09; 0.40]). Most arms demonstrated a statistically significant reduction in the incidence of overt HE compared to albumin and placebo. The results also demonstrated a significant reduction in ammonia between L-ornithine-L-aspartate (LOLA) and probiotics (MD= -19.17, 95% CI [-38.01; -0.32]), as well as a significant difference in the incidence of LOLA compared to placebo (MD= -22.62, 95% CI [-39.16; -6.07]). This network meta-analysis has significant data for managing subclinical HE in people without a history of overt HE. Our analysis showed that (Lactulose and Rifaximin), followed by (Rifaximin and L-carnitine), followed by (Lactulose and Rifaximin with zinc) were the best combinations regarding overt HE. LOLA reduced ammonia best, followed by Nitazoxanide and finally Lactulose. (Lactulose and Nitazoxanide) have the least adverse effects, followed by (Rifaximin and L-carnitine), then Probiotics. Yet, all mortality outcomes and quality of life changes yielded no useful findings. Future studies like RCTs must be done to compare our therapies directly.

## Introduction and background

Cirrhosis is the 14th most common cause of death worldwide [1], and the 12th most common cause of death in the United States [2], which represents a challenge for healthcare providers to extend the patient’s life without going through liver transplantation. Under physical stress, patients with liver cirrhosis have a lower ventricular ejection fraction than non-cirrhotic subjects. Hyperdynamic circulation, cirrhotic cardiomyopathy, and pulmonary vascular abnormalities are just some of the cardiovascular abnormalities linked to liver cirrhosis. Moreover, cirrhosis can occur with hepatic encephalopathy (HE) due to the brain accumulation of ammonia and manganese accompanied by inflammation [3].

HE refers to brain dysfunction caused by liver insufficiency and/or portal-systemic blood shunting [4]. In both chronic liver disease and acute liver failure, HE is a common and potentially fatal complication [5]. The mildest form of HE is called minimal HE, and it's linked to forgetfulness and an inability to focus. The mild HE that can develop from the minimal HE causes alterations in mood and sleep patterns. Mild HE has the potential to progress to moderate HE, which can have an impact on the patient's personality and behavior in addition to their slurred speech and mathematical difficulties. Disorientation, prolonged periods of sleep, coma, and even death can result from untreated severe HE if the case becomes more complex. Patients with chronic liver disease but no obvious HE have a reported prevalence of 30%-84% for minimal HE. The main way to treat HE is the reduction in the ammonia level in the blood [6]. Minimal HE is the highest incidence as it ranges from 30-84% in patients with chronic liver disease without overt HE [7, 8]. Minimal HE is easily underestimated due to the word minimal as a prefix, although previous studies have reported high rates of deaths due to incorrect diagnoses of minimal HE as well as the damages that minimal HE may cause to society [8], as patients with minimal HE were reported to have a higher crash rate due to accidents than the normal population due to the effects of minimal HE on impairing driving skills and reducing conscious awareness [9]. There are a lot of treatment options to reduce the high level of ammonia in blood as Lactulose, Rifaximin, branched-chain amino acids (BCAA), L-ornithine-L-aspartate (LOLA), and Probiotics. In our network meta-analysis we are investigating the multiple interventions to treat the different types of HE.

## Review

Methods

We conducted our study in substantial accordance with the Cochrane handbook guidelines for systematic reviews of interventions, then we reported according to the preferred reporting items for systematic reviews and meta-analyses (PRISMA) [10, 11].

Search Strategy and Data Collection

(albumin OR probiotics OR prebiotics OR lactobacillus OR bifidobacterium OR symbiotics OR lactulose OR lactitol OR disaccharides OR rifaximin OR rifagut* OR xifaxan* OR rcifax* OR “branched chain amino acid” OR “branched chain amino acids” OR “L-ornithine L-aspartate” OR LOLA OR BCAA OR BCAAs OR “branched chain” OR “amino acids”)AND (encephalopathy OR cirrhosis OR “minimal hepatic encephalopathy” OR “subclinical hepatic encephalopathy” OR “latent hepatic encephalopathy” OR “covert hepatic encephalopathy” OR “liver cirrhosis”)

Selection Criteria

We considered studies that met the following requirements: (1) Population: Cirrhotic individuals or patients at risk for hepatic encephalopathy; (2) Intervention: Lactulose, rifaximin, nitazoxanide, probiotics, L-ornithine-L-aspartate, Rifaximin and L-carnitine, lactitol, metronidazole, polyethylene glycol (PEG), Rifaximin and L-carnitine, Lactulose and Nitazoxanide, and Lactulose and Rifaximin; (3) Comparator: placebo or control; (4) Outcomes: change in health-related quality of life, reduction in ammonia, adverse events, all-cause of mortality, development of overt HE, reversal of minimal HE; (5) Study design: RCTs and observational studies.

Data Extraction

We extracted the data related to the following: Summary of the included studies, including inclusion criteria, study design, follow-up, study groups, etiology of cirrhosis, and conclusion. Baseline characteristics of the enrolled population, including sample size, age, study ID, gender, site, ammonia, MELD score, and outcomes of change in health-related quality of life, reduction in ammonia, adverse events, all causes of mortality, development of overt HE, reversal of minimal HE.

Quality Assessment

We used Cochrane's risk of bias tool (version 1) to assess the included interventional studies' quality. The tool is reported in chapter 8.5 of the Cochrane Handbook for Systematic Reviews of Interventions 5.1.0. The tool consists of the following assessment items: sequence generation, allocation sequence concealment, blinding of participants and personnel, blinding of outcome assessors, incomplete outcome data, selective outcome reporting, and any other bias; author judgments fall into three categories; low, unclear or high risk of bias for each item. We used the quality assessment table in part 2, Chapter 8.5 of the same book [12]. We used the tool of observational cohort studies, which is composed of questions assessing the risk of bias and confounders. Each question was answered by “yes”, “no”, “not applicable”, “not reported”, and “cannot determine”, then each study was given a score to guide the overall quality either “poor”, “fair”, or “good”.

Statistical Analysis

Using frequentist network meta-analysis with random-effects models, the continuous data were pooled as mean difference (MD) and their 95% confidence interval (CI), while the binary data was extracted as risks ratios (RRs). MetaInsight Version 3.14, an interactive web-based tool for network meta-analyses based on the R-shiny and netmeta statistical packages, was used for all statistical analyses. Heterogeneity was assessed under clinical, methodological, and statistical domains. Statistical heterogeneity was assessed using the I2 statistics [13].

Results

Literature Search

**Figure 1:**
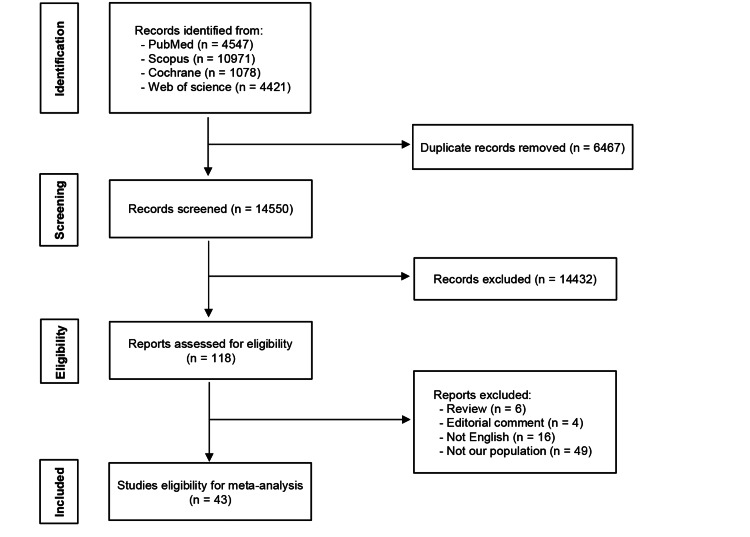
PRISMA Flow diagram PRISMA 2020 flow diagram for new systematic reviews which included searches of databases and registers only.

The initial results after searching our main four databases were 21017 results, with the help of the EndNote program we removed 6467 duplicates then we did a title and abstract screening for 14550 results. The final result was to include 43 studies [14-56]. The full PRISMA is presented in Figure 1.

Included Population's Baseline Characteristics & Included Studies' Summary

Our study included 32 randomized clinical trials (RCTs) and 10 cohort studies, both retrospective and prospective, as well as one case-control study. Numerous international and/or multicenter studies contributed to the global scope of the included studies. We included patients from various nations, including Germany, the United States, Japan, Egypt, China, and Taiwan. The full baseline and summary characteristics are presented in Table 1.

**Table 1:** Baseline and Summary HBV: Hepatitis B Virus, HCV: Hepatitis C Virus, NAFLD: Non-Alcoholic Fatty Liver Disease, AIH: Autoimmune Hepatitis, PBC: Primary Biliary Cholangitis, NBNC‐NA: Non-B, Non-C Non-Alcoholic, AILDs: Autoimmune Liver Diseases, DRLDs: Drug-Induced Liver Diseases, AC: Alcoholic Cirrhosis, NASH: Non-alcoholic steatohepatitis, LOLA: L-ornithine-L-aspartate, BCAA: branched-chain amino acids, HRQOL: Health-related quality of life. References: [14-56]

N	Study ID	Study arms, n(%)	Site	Study design	Age,(M±SD)y	Male, n(%)	Follow-up, month	Etiology of cirrhosis, n(%)	Child-Pugh class, n(%)	MELD score,(M±SD)	Ammonia, μmol/L (M±SD)	Inclusion criteria	Main outcomes	Conclusion
1	Abd-Elsalam, 2018 [14]	Nitazoxanide and Lactulose, 60(50)	Egypt	RCT	58.16 ± 8.45	38(63.3)	Until 10 days after the end of treatment	NR	1. B, 21(46.7) 2. C, 39(53.3)	NR	NR	1. Patients suffering from LC and overt HE. 2. All participants or their relatives signed informed written consents. 3. During a time frame of 6 months starting January 2016.	CHESS score	"NTZ significantly decreases the CHESS score and improves mental status in the form of patient alertness, orientation, response to stimulation, and the ability to talk. NTZ is safe and well tolerated apart from infrequent epigastric pain"
		Lactulose, 60(50)			55.66 ± 6.62	30(50.0)			1. B, 14(23.3), 2. c, 46(76.7)					
2	Alvares-da-silva, 2013 [15]	LOLA, 28(44.44)	Brazil	RCT	51.3 ± 13.5	15(53.6)	Six	NR	A/B, 25(89.3)	12 ± 13.5	49.5 ± 19.9	Adult cirrhotic outpatients with MHE defined by psychometric tests: number connection tests A and B (NCT-A/B) and digit	1. OHE 6 months 2. Child-Pugh 3. Reduction in Ammonia	"A 60-day oral LOLA course was not better than a placebo in treating MHE but was useful in preventing further episodes of OHE"
		Placebo, 35(55.56)			52.5 ± 11.5	17(48.6)			A/B, 28(80)	12 ± 13.1	47.7 ± 35.17			
3	Bai, 2019 [16]	Albumin 30g, 354(50)	Multicenter	Retrospective cohort study	58.12±12.57	226(63.80)	NR	1. HBV, 192(27.1), 2. HCV, 43(6.10), 3. Alcohol Abuse(AA), 150(21.20), 4. HBV and AA, 68(9.60), 5. HCV and AA, 11(1.60), 6. DRLDs, 9(1.30), 7. AILDs, 46(6.50), 8. Others, 189(26.70)	7.97 (SD 1.69)	8.10±6.03	45.79±71.75	1. Between January 2010 and June 2014, 2. cirrhotic patients without overt HE.	1. Improvement of overt HE, 2. In-hospital Death	"Based on the results of our retrospective study and meta-analysis, albumin infusion might prevent the occurrence of overt HE and improve the severity of overt HE in cirrhosis. Our retrospective study also suggested that albumin infusion improved the outcomes of cirrhotic patients regardless of overt HE".
		Control, 354(50)			57.52±12.52	214(60.50)			7.96 (SD 1.74)	8.51±7.41	48.62±42.26			
4	Bai, 2019-2 [17]	ALB > 31.6g/l, 1638(69.02)	China	Retrospective cohort study	54.80 ± 12.18	1119(68.3)	Six to 60	1. HBV, 920(38.7), 2. HCV, 193(8.1), 3. Alcohol abuse, 904(38), 4. Autoimmune, 147(6.2), 5. Others, 551(23.2)	6.14(SD 1.27)	5.19 ± 5.43	38.17 ± 28.07	1. From January 2010 to June 2014, 2. Cirrhotic patients without malignancy	1. Improvement of Overt HE, 2. In-hospital Death, 3. ALB level	"Decreased serum ALB level may be associated with a higher risk of overt HE and HE-associated mortality during hospitalizations in cirrhosis"
		ALB ⩽ 31.6 g/l, 738(31.06)			56.30 ± 11.80	500(67.8)			8.02(SD 1.59)	8.06 ± 6.42	51.26 ± 43.85			
5	Bajaj, 2011 [18]	Rifaximin, 21(50)	USA	RCT	55 ± 5	NR	Two	Alcoholic cirrhosis, 5(21)	Child score, A/B/C, 19/2/0	Median (9)	49 ± 28	1. Patients had never had a prior overt HE. 2. Were not on therapy for it, were not on any psychoactive medications. 4. With cirrhosis and minimal HE	1. Reduction in Ammonia. 2. Safety and adverse events	"Patients with MHE significantly improve driving simulator performance after treatment with rifaximin, compared with placebo"
		Placebo, 21(50)			57 ± 5			Alcoholic cirrhosis, 3(14)	Child score, A/B/C, 17/4/0	Median (9)	43 ± 20			
6	Bajaj, 2014 [19]	LGG, 18(48.65)	USA	RCT	56.3 ± 9	12(66.67)	Two	1. HCV, 8(44.44), 2. HCV and alcohol, 3(16.67), 3. Alcohol, 1(5.55) 4. NASH, 3(16.67) 5. Others, 3(16.67)	NR	8.6 ± 2.2	NR	1. Patients with cirrhosis 2. Had been stable for 6 months without treatment. 3. Between the age range 18–65y	1. Reduction in Ammonia. 2. Safety and adverse events. 3. Serious adverse events.	"In this phase I study, Lactobacillus GG is safe and well-tolerated in cirrhosis and is associated with a reduction in endotoxemia and dysbiosis"
		Placebo, 19(51.35)			58.4 ± 4.3	13(68.42)		1. HCV, 8(42.1) 2. HCV and alcohol, 9(47.36) 3. Alcohol, 0 4. NASH, 6(31.57) 5. Others, 3(15.158)		8.3 ± 2				
7	Bajaj, 2019 [20]	Lactulose, 695(24.73)	Multicentre in the USA	Prospective Cohort Study	57.23 ± 10.11	431(62)	Three	1. AC, (37) 2. HCV, (19) 3. HCV and AC, (15) 4. NASH, (17) 5. Other, (12)	9.86 (SD 2.08)	19.41 ± 7.65	NR	1. Patients with cirrhosis. 2. Hospitalised for non‐elective reasons. 3. From April 2013 through February 2017. 4. All patients gave written informed consent	1. ICU transfer 2. In‐hospital mortality. 3. In‐hospital transplant	"Several targets to improve HE management were identified in a large cohort of hospitalized cirrhotic patients. Interventions to decrease medication-precipitated HE, prevention of aspiration pneumonia, and optimization of HE medications are warranted"
		Rifaximin, 154(5.48)			57.22 ± 11.28	86(54)		1. AC, (23) 2. HCV, (23) 3. HCV and AC, (15) 4. NASH, (25) 5. Other, (20)	9.97 (SD 1.93)	20.15 ± 7.33				
		Lactulose and Rifaximin, 895(31.85)			57.30 ± 9.42	555(62)		1. AC, (29) 2. HCV, (22) 3. HCV and AC, (15) 4. NASH, (24) 5. Other, (11)	10.24 (SD 1.97)	21.30 ± 7.66				
		Control, 1102(39.21)			57.21 ± 12.03	695(63)		1. AC, (30) 2. HCV, (20) 3. HCV and AC, (13) 4. NASH, (21) 5. Other, (17)	8.88 (SD 2.18)	18.03 ± 7.50				
8	Bajaj, 2022 [21]	Rifaximin SSD IR 40mg, 78(15.11)	USA	RCT	56.4 ± 10.3	52(66.7)	Seven	1. Viral hepatitis only, 27(34.6) 2. Alcohol-induced only, 23(29.5) 3. Both, 9(11.5) 4. Other, 19(24.4)	1. Class A, 10(12.8) 2. Class B, 64(82.1) 3. Class C, 4(5.1)	11.5 ± 0.3	NR	1. Aged 18 years 2. All patients gave written informed consent 3. Patients with cirrhosis 4. With sodium (MELD-Na) score >=12	1. Reduction in Ammonia 2. All-cause mortality 3. Safety and adverse events	"Rifaximin SSD IR 40 mg may reduce hospitalizations in patients with cirrhosis and shorten the duration of OHE during hospitalization—considered a negative finding, yet also hypothesis-generating"
		Rifaximin SSD IR 80mg, 91(17.63)			56.9 ± 9.1	52(57.1)		1. Viral hepatitis only, 29(31.9) 2. Alcohol-induced only, 22(24.2) 3. Both, 16(17.6) 4. Other, 24(26.4)	1. Class A, 11(12.1) 2. Class B, 74(81.3) 3. Class C, 6(6.6)	11.6 ± 0.4				
		Rifaximin SSD SER 40mg, 84(16.28)			57.4 ± 9.3	43(51.2		1. Viral hepatitis only, 19(22.6) 2. Alcohol-induced only, 19(22.6) 3. Both, 8(9.5) 4. Other, 38(45.2)	1. Class A, 16(19.0) 2. Class B, 62(73.8) 3. Class C, 6(7.1)	10.9 ± 0.4				
		Rifaximin SSD SER 80mg, 89(17.25)			57.5 ± 9.3	56(62.9)		1. Viral hepatitis only, 32(36.0) 2. Alcohol-induced only, 15(16.9) 3. Both, 15(16.9) 4. Other, 27(3.3)	1. Class A, 6(6.7) 2. Class B, 77(86.5) 3. Class C, 6(6.7)	11.5 ± 0.4				
		Rifaximin SSD IR 80mg and SER 80mg, 80(15.5)			57.2 ± 9.1	51(63.8)		1. Viral hepatitis only, 26(32.5) 2. Alcohol-induced only, 19(23.8) 3. Both, 10(12.5) 4. Other, 25(31.3)	1. Class A, 8(10.0) 2. Class B, 62(77.5) 3. Class C, 10(12.5)	12.1 ± 0.4				
		Placebo, 94(18.21)			57.4 ± 8.6	61(64.9)		1. Viral hepatitis only, 27(28.7) 2. Alcohol-induced only, 27(28.7) 3. Both, 16(17.0) 4. Other, 24(25.5)	1. Class A, 10(10.6) 2. Class B, 77(81.9) 3. Class C, 7(7.4)	11.5 ± 0.4				
9	Chang, 2021 [22]	Rifaximin and Lactulose, 12(27.9)	Taiwan	Retrospective cohort study	67 ± 7.95	6(50)	12	NR	NR	14.1567 ± 3.92	40.24 ± 11.35	1. From January 2015 to December 2019 2. Patients age 18 years or older 3. Diagnosed with liver cirrhosis complicated by HE	1. HE recurrence-free rate 2. All-cause mortality 3. Reduction in Ammonia 4. Safety and adverse events	"The above results provide evidence for the one-year use of rifaximin add-on to lactulose in reducing HE recurrence and HE-related hospitalization for patients with decompensated cirrhosis"
		Lactulose, 31(72.1)			57.58 ± 12.28	20(64.5)				17.67 ± 6.217	42.1 ± 19.8			
10	Dhiman, 2000 [23]	Lactulose, 14(35)	India	RCT	44.1 ± 18.0	13(92.85)	Three	1. Alcohol, 5(35.71) 2. HBV, 2(14.28) 3. Alcohol and HBV, 1(7.14) 4. Autoimmune, 1(7.14) 5. Others, 5(35.71)	1. Child A, 5(35.71) 2. Child B, 6(42.85) 3. Child C, 3(21.42)	NR	NR	1. Cirrhotic patients 2. clinically stable, and none had OHE	Changes in the mean number of normal psychometric test results	"We conclude that lactulose treatment in cirrhotic patients with SHE is effective"
		Control, 12(30)			47.8 ± 13.5	8(66.67)		1. Alcohol, 3(25) 2. HBV, 4(33.33) 3. Alcohol and HBV, 1(8.33) 4. Others, 4(33.33)	1. Child A, 3(25) 2. Child B, 5(41.67) 3. Child C, 4(33.33)					
		NSHE, 14(35)			39.2 ± 9.6	12(85.71)		1. Alcohol, 7(50) 2. HBV, 3(21.42) 3. Autoimmune, 1(7.14) 4. Others, 3(21.42)	1. Child A, 5(35.71) 2. Child B, 6(42.85) 3. Child C, 3(21.42)					
11	Sidhu, 2011 [24]	Rifaximin, 49(52.13)	India	RCT	51.67 ± 4.05	40(81.63)	Two	1. Alcohol, 27(55.1) 2. HBV, 0 3. HCV, 19(38.77) 4. Other, 5(10.2)	1. A, 14(28.57) 2. B, 31(63.26) 3. C, 4(8.16)	NR	NR	1. Patients with liver cirrhosis without OHE 2. Age 18–65 years	1. Reversal of MHE 2. Total SIP score	"Rifaximin significantly improves both cognitive functions and HRQOL in patients with MHE"
		Placebo, 45(47.87)			54.33 ± 4.21	34(75.55)		1. Alcohol, 21(46.67) 2. HBV, 1(2.22) 3. HCV, 20(44.44) 4. Other, 4(8.88)	1. A, 16(35.56) 2. B, 23(51.11) 3. C, 5(11.11)					
12	Feuerstadt, 2019 [25]	Rifaximin, 149(21.26)	USA	Retrospective Case-control study	59.3 ± 9.6	87(58.4)	NR	NR	NR	19.7 ± 9.8	NR	1. Patients with cirrhosis and Symptomatic diarrhea 2. On chronic rifaximin therapy 3. Between January 1, 2010, and December 31, 2014	1. All-cause mortality 2. ICU requirement	"Patients with cirrhosis who are on chronic rifaximin have decreased rates of CDI compared with those not on this therapy. Despite its risk for promoting resistance, chronic rifaximin use may have a beneficial effect in preventing CDI in patients with cirrhosis"
		Control, 552(78.74)			62.2 ± 11.7	230(54.3)				15.5 ± 8.1				
13	Flamm, 2018 [26]	Rifaximin, 140(46.82)	USA	RCT	55.5 ± 9.6	75(53.6)	Six	NR	NR	13.1 ± 3.6	NR	1. Adults with cirrhosis 2. History of ⩾2 episodes of overt HE 3. With a MELD score ⩽of 25 at the study entry	1. Patients free from cirrhosis complications 2. Patients free from non-HE complications of cirrhosis	"Rifaximin may reduce the incidence of cirrhosis-related complications and the recurrence of overt HE"
		Placebo, 159(53.12)			56.8 ± 9.2	107(67.3)				12.7 ± 3.9				
14	Fritz, 2022 [27]	Lactulose and Rifaximin, 55(35.03)	USA	Retrospective cohort study	59.5 ± 8.2	38 (69.1)	12	1. Alcohol, 11(20.0) 2. HCV, 7(12.7) 3. NASH, 32(58.2) 4. Combination, 3(5.5) 5. Other, 2(3.6)	NR	19 ± 5.7	NR	1. From July 2016 through June 2019 2. 18 to 80 years of age 3. With a documented history of cirrhosis	1. All-cause readmissions 2. HE readmissions 3. HE readmissions	"This is the first study conducted in the United States evaluating zinc for HE treatment. Zinc did not impact 30-day or 1-year all-cause readmission rates. Further studies are warranted to evaluate the potential benefit of zinc for HE, possibly in correlation with Model for End-stage Liver Disease-Sodium (MELD-Na) scores"
		Control, 102(64.97)			56.9 ± 10.4	55 (53.9)		1. Alcohol, 14(13.7) 2. HCV, 11(10.8) 3. NASH, 54(52.9) 4. Combination, 14(13.7) 5. Other, 9(8.8)		18.4 ± 6.6				
15	Glal, 2021 [28]	Rifaximin, 30(50)	Egypt	RCT	54.4 ± 7.6	17 (56.7)	Six	1. HCV, 28(93.3) 2. HBV, 2(6.7)	10.4 (SD 1.7)	16.4 ± 6.6	81.6 ± 22.9	1. Egyptian cirrhotic adult patients 2. 18 to 80 years of age 3. Age range between 20 and 65y old 4. Had at least one previous episode of HE.	1. All-cause readmissions 2. Reduction in Ammonia 3. Improvement in CLDQ	"Nitazoxanide may represent a suitable and safe alternative therapy to rifaximin in preventing the recurrence of hepatic encephalopathy"
		Nitazoxanide, 30(50)			54.6 ± 6.5	19 (63.4)		1. HCV, 29(96.7) 2. HBV, 1(3.3)	9.6 (SD 1.4)	16.9 ± 5.4	83.9 ± 23.2			
16	Hiramine, 2021 [29]	Rifaximine, 76(100)	Japan	Retrospective cohort study	68.5 ± 9.5	41(53.95)	24	1. HBV, 5(6.58) 2. HCV, 23(30.26) 3. Alcohol, 21(27.63) 4. NBNC‐NA, 15(19.73) 5. Other, 12(15.79)	1. A, 2(2.63) 2. B, 44(57.89) 3. C, 30(39.5)	NR	32.31 ± 8.74	1. From November 2016 to December 2019 2. Patients with HE and accompanying hyperammonemia 3. Developed OHE of West Haven grade II or higher	1. All-cause readmissions 2. Reduction in Ammonia 3. Incidence of overt OHE	"Rifaximin was associated with decreased blood ammonia levels, lower incidence of OHE, and fewer hospitalizations in Japanese patients with HE. In addition, serum albumin level was an important predictor of the efficacy of rifaximin"
17	Horsmans, 1997 [30]	Lactulose, 7(50)	Belgium	RCT	59 ± 8.7	3(42.86)	2 weeks	1. Alcoholic, 2(28.57) 2. Non-Alcoholic, 3(42.86)	NR	NR	NR	1. Patients with Alcoholic and non-alcoholic cirrhosis 2. Stable clinical condition 3. Presence of oesophageal varices 4. Absence of apparent Clinical encephalopathy 5. Normal ECG	1. Psychometric testing expressed mean changes from initial values after administration of Lactulose and Placebo 2. Reduction in Ammonia	"Our data suggest that Lactulose therapy might improve subclinical hepatic encephalopathy in patients with cirrhosis and portal-systemic shunting"
		Placebo, 7(50)			56.1 ± 14.2	4(57.14)		1. Alcoholic, 5(71.43) 2. Non-Alcoholic, 4(57.14)						
18	Jain, 2021 [31]	LOLA, 67(50)	India	RCT	43.91 ± 11.13	58(86.57)	One	1. Alcohol, 46(68.66) 2. HBV, 13(19.4) 3. HCV, 2(2.99) 4. AIH, 2(2.99) 5. Others, 4(59.4)	12.51 (SD 2.17)	23.46 ± 5.46	99.25 ± 11.93	1. Written informed consent 2. Patients aged 18–70 years 3. With a diagnosis of cirrhosis 4. With overt grade III– IV HE	1. Change in ammonia 2. Change in Inflammatory markers 3. All-cause mortality	"Combination of LOLA with lactulose and rifaximin was more effective than only lactulose and rifaximin in improving grades of HE, recovery time from encephalopathy, with lower 28-day mortality"
		Placebo, 67(50)			44.51 ± 9.03	53(79.1)		1. Alcohol, 47(70.15) 2. HBV, 1(1.49) 3. HCV, 10(14.93) 4. AIH, 7(10.45) 5. Others, 2(2.99)	12.43 (SD 1.34)	24.12 ± 6.29	101.19 ± 11.627			
19	Jain, 2013 [32]	Lactulose, 30(37.5)	India	RCT	42 ± 13.75	20(66.67)	Three	1. Alcohol, 18(60) 2. HBV, 5(16.6) 3. HCV, 4(13.4)	1. A, 12(40) 2. B, 12(40) 3. C, 6(20)	19 ± 5	NR	1. Patients with cirrhosis 2. From October 2011 to February 2012	1. Change in Ammonia 2. Change in Inflammatory markers	"Arterial ammonia, inflammatory mediators (TNF-a, IL-6, IL-18), and serum endotoxin reduces and MRS abnormalities improve after treatment with lactulose in patients with MHE"
		Control, 30(37.5)			41 ± 13	19(63.33)		1. Alcohol, 17(56.6) 2. HBV, 6(20) 3. HCV, 5(16.6)	1. A, 14(46.67) 2. B, 12(40) 3. C, 4(13.33)	20 ± 4.5				
		No MHE, 20(25)			41.25 ± 12.25	18(60)		1. Alcohol, 12(60) 2. HBV, 4(20) 3. HCV, 3(15)	1. A, 9(45) 2. B, 8(40) 3. C, 3(15)	21 ± 4.5				
20	Kircheis, 1997 [33]	LOLA, 63(50)	Germany	RCT	53.9 ± 12.4	45(71)	7 days	1. Alcohol, 49(78) 2. Posthepatitic, 12(19) 3. Others, 2(3)	1. A, 30(48) 2. B, 28(44) 3. C, 5(8)	NR	NR	1. Chronic (persistent), manifest HE 2. Hyperammonemia (venous ammonia concentration œ50mmol/L	1. NCT-A performance over times 2. Change in Ammonia levels} 3. Incidence of Overt HE	"OA infusion appears to be a safe, effective treatment of chronic (persistent) manifest HE in cirrhotic patients. Additional investigations are required to assess the efficacy of OA in patients with SHE, as well as in patients with more severe grades of HE"
		Placebo, 63(50)			52.3 ± 13.3	46(73)		1. Alcohol, 51(81) 2. Posthepatitic, 8(13) 3. Others, 4(6)	1. A, 34(54) 2. B, 22(35) 3. C, 7(11)					
21	Kubota, 2021 [34]	Rifaximin, 42(50.6)		RCT	65.6 ± 11.6	29(69)	Three	1. Virus, 8(19.5) 2. ALC, 13(31) 3. NASH, 14(33.33) 4. etc, 7(16.67)	8.46 (SD 1.5)	NR	22.66 ± 8.771	1. Grade I or II HE 2. One or more occurrences of overt HE 3. History of malignancies other than HCC 4. Severe renal and/or HF	1. Change in Laboratory data 2. Adverse events	"L-carnitine addition reduced the risk of hospitalization for patients who received rifaximin for HE"
		L-carnitine and Rifaximin, 41(49.4)			68.0 ± 10.5	28(68.3)		1. Virus, 7(17.7) 2. ALC, 13(32) 3. NASH, 12(29.27) 4. etc, 9(21.95)	8.67 (SD 1.6)		23.4 ± 7			
22	Lunia, 2014 [35]	Probiotics, 86(53.75)	India	RCT	48.5 ± 10.5	86(60.47)	9.825 ± 2.325	1. Alcohol, 42(48.8) 2. HBV, 18(20.9) 3. HCV, 6(7) 4. Cryptogenic, 15(17.4) 5. Other, 5(5.8)	9.78(SD 2.53)	19.85 ± 5.18	74.3 ± 18.6	1. From January 2012 to March 2013 2. Patients between the ages of 18 and 75 years 3. With cirrhosis and no previous history of HE	1. Incidence of HE 2. Change in Ammonia levels	"In a prospective, randomized controlled trial, probiotics were found to be effective in preventing HE in patients with cirrhosis"
		Control, 74(46.25)			49.4 ± 11.5	44(59.46)		1. Alcohol, 40(54.1) 2. HBV, 13(17.6) 3. HCV, 5(6.8) 4. Cryptogenic, 13(17.6) 5. Other, 3(4.1)	9.68(SD 3.16)	18.94 ± 6.24	78.4 ± 15.6			
23	Mekky, 2018 [36]	Metronidazole, 60(50)	Egypt	RCT	51 ± 11	45(75)	NR	NR	1. A 0 2. B 13(21.67) 3. C 47(78.33)	20.5 ± 6.3	NR	1. Between January 2017 and August 2017 2. With an acute episode of OHE on top of cirrhosis	1. Clinical improvement of HE 2. Changes in the level of serum ammonia	"Rifaximin and metronidazole are equally effective in the management of an acute episode of overt HE, therefore, re-auditing of treatment protocols of HE is warranted especially in limited resource settings"
		Rifaximin, 60(50)			49 ± 12	50(83.33)			1. A 0 2. B 9(15) 3. C 51(85)	21.8 ± 5.5				
24	Mittal, 2011 [37]	Lactulose, 40(25)	India	RCT	43.85 v 10.9	32(80)	Three	1. ALD, 17(42.5) 2. Viral, 14(35) 3. Others, 9(22.5)	8 (SD 1.75)	16.71 ± 4.0	80.02 ± 15.5	Patients with cirrhosis with MHE	1. MHE Improvement 2. OHE Incidence 3. Change in Ammonia levels	"Lactulose, probiotics, and LOLA significantly improve MHE and HRQoL in patients with chronic liver disease"
		Probiotics, 40(25)			44.25 ± 11.8	30(75)		1. ALD, 18(45) 2. Viral, 13(32.5) 3. Others, 9(22.5)	8 (SD 1.75)	17.59 ± 3.9	80.48 ± 20.3			
		LOLA,40(25)			42.15 ± 8.7	31(77.5)		1. ALD, 17(42.5) 2. Viral, 14(35) 3. Others, 9(22.5)	7.5 (SD 1.75)	17.7 ± 3.9	75.13 ± 20.2			
		Control, 40(25)			41.2 ± 11.9	30(75)		1. ALD, 14(35) 2. Viral, 14(35) 3. Others, 12(30)	8 (SD 1.75)	17.04 ± 4.2	76.88 ± 15.8			
25	Miwa, 2022 [38]	Rifaximin, 8(100)	Japan	Prospective Cohort Study	70 ± 8	3(37.5)	Three	NR	6 ± 1	11 ± 4	25.6 ± 8	1. Patients with LC of any etiology 2. Aged between 20 and 79 years 3. HE grades I or II	1. HBC, 1(12.5) 2. HCV, 1(12.5) 3. ALD, 2(25) 2. NALD, 2(25) 5. Others, 2(25)	"Thus, rifaximin reduces serum ammonia levels and may improve circulating albumin structure in patients with cirrhosis. Further large-scale studies are required to confirm these preliminary results"
26	Moneim, 2021 [39]	Rifaximin and Lactulose, 12(27.9)	Egypt	RCT	58.46 ± 7.75	30(60)	Six	NR	1. Class B, 14(28) 2. Class C, 36(72)	a. ≤10, 7(14) b. 11-18, 34(68) c. 19-24, 9(18) d. ≥25, 0	NR	1. Between January 2015 and Dec 2018 2. Cirrhosis because of HCV infection 3. Age 18 to 75 years 4. MELD score ≤ 25	1. HE devolopement 2. OHE Incidence 3. MIC change	"Rifaximin succeeded to maintain remission from new episodes of HE in hepatitis C virus cirrhotic patients with limited potential for development of microbial resistance over the study period"
		Control			60.50 ± 7.63	29(58)			1. Class B, 15(30) 2. Class C, 35(70)	a. ≤10, 4(8) b. 11-18, 33(66) c. 19-24, 13(26) d. ≥25, 0				
27	Mouli, 2015 [40]	Lactulose, 60(50)	India	RCT	15 to 80y	53(88.3)	Two	1. Alcohol, 21(35) 2. Viral, 24(40) 3. Others, 15(25)	8 (SD 11.9)	13.11 ± 5.48	79.4 ± 9.025	1. Diagnosis of MHE in patients with cirrhosis 2. Aged between 15 and 80 years	1. MHE improvement 2. Change in ammonia levels	"The probiotic VSL#3 was non-inferior to the standard therapy, lactulose in the treatment of MHE. Improvement in MHE correlated with reduction of ammonia levels"
		Probiotics, 60(50)				57(95)		1. Alcohol, 24(40) 2. Viral, 24(40) 3. Others, 12(20)	8.5 (SD 12.2)	14.28 ± 5.56	96.6 ± 131			
28	Okada, 2020 [41]	Lactulose, 491(6.1)	Japan	Retrospective cohort study	a. ≤64y, 135(27.5) b. 65–74y, 153(31.2) c. ≥75y, 203(41.3)	248(50.5)	NR	NR	1. Class A, 8(1.6) 2. Class B, 135(27.5) 3. Class C, 348(70.9)	NR	NR	1. Diagnosis of HE or liver diseases 2. From 1 July 2010 to 31 March 2017	1. Mortality rate 2. Impaired mental status at discharge	"Branched-chain amino acid infusion together with lactulose may improve the prognosis of hepatic encephalopathy"
		Lactulose and BCAA,7560(93.9)			a. ≤64y, 1875(24.8) b. 65–74y, 2555(33.8) c. ≥75y, 3130(41.4)	3931(52)			1. Class A, 159(2.1) 2. Class B, 2722(36) 3. Class C, 4680(61.9)					
29	Plauth, 1993 [42]	BCAA, 9(52.94)	Germany	RCT	52 ± 10	6(66.67)	Two	1. Alcohol 7(77.78) 2. Viral hepatitis 2(22.22)	1. A, 2(22.22) 2. B, 6(66.67) 3. C, 1(11.11)	NR	56 ± 4	1. Between June 1983 and June 1986 2. Patients (<65 years) 3. With clinically proven cirrhosis	1. Improvement in Psychomotor disturbance 2. Change in nutritional parameters	"We conclude that long-term branched-chain amino acid supplementation is well tolerated and effective in the treatment of impaired automobile driving capacity associated with latent portosystemic encephalopathy"
		Placebo, 8(47.06)			49 ± 14	5(62.5)		Alcohol, 8(100)	1. A, 3(37.5) 2. B, 4(50) 3. C, 1(12.5)		57 ± 2			
30	Prasad, 2007 [43]	Lactulose, 31(34.44)	India	RCT	48.3 ± 4.95	27(87.1)	Three	1. Alcohol, 20(64.5) 2. HBV, 6(19.3) 3. HCV, 3(9.7) 4. Other, 2(6.5)	1. A, 10(32.3) 2. B, 16(51.6) 3. C, 5(16.1)	NR	NR	Patients diagnosed as having cirrhosis	1. Reduction in Ammonia 2. Change in HRQOL 3. Improvement in MHE	"Treatment with lactulose improves both cognitive function and HRQOL in patients with cirrhosis who have MHE"
		Control, 30(33.33)			50.6 ± 5.75	28(93.33)		1. Alcohol, 20(66.7) 2. HBV, 5(16.7) 3. HCV, 4(13.3) 4. Other, 1(3.3)	1. A, 10(33.3) 2. B, 17(56.7) 3. C, 3(10)					
		NMHE, 29(32.22)			45.4 ± 3.8	25(86.21)		1. Alcohol, 17(58.6) 2. HBV, 6(20.8) 3. HCV, 4(13.8) 4. Other, 2(6.8)	1. A, 13(44.8) 2. B, 12(41.8) 3. C, 4(13.8)					
31	Rattanasupar, 2021 [44]	Lactulose, 24(52.17)	Thailand	RCT	51.9 ± 14.7	19(79.2)	NR	NR	8 (SD 1.7)	15.0 ± 4.7	NR	1. Patients with liver cirrhosis 2. Aged 18–80 years 3. From October 2012 to February 2014 4. Without HE at the time of admission	1. Adverse events 2. OHE development 3. Mortality rate	"Five-day lactulose is ineffective as prophylaxis against HE in cirrhotic patients with AUGIB. Unnecessary treatment with laxatives should be avoided in these patients"
		Placebo, 22(47.83)			49.7 ± 14.2	19(86.4)			8.8 (SD 2.4)	15.6 ± 6.4				
32	Sharma, 2008 [45]	Lactulose, 35(33.33)	India	RCT	39.5 ± 13	NR		NR	1. A, 14(40) 2. B, 11(31.43) 3. C, 10(28.57)	NR	NR	1. From February 2005 to August 2006 2. Cirrhotic patients without OHE	1. Reduction in Ammonia 2. Response to treatment	"A total of 55% of the patients with cirrhosis had MHE. Lactulose or probiotics or combinations of both are equally effective in the treatment of MHE"
		Probiotics, 35(33.33)			43.5 ± 12.1				1. A, 12(34.29) 2. B, 14(40) 3. C, 9(25.71)					
		Lactulose and Probiotics, 35(33.33)			43.7 ± 10				1. A, 10(28.57) 2. B, 14(40) 3. C, 11(31.43)					
33	Sharma, 2014 [46]	LOLA, 31(25)	India	RCT	42 ± 11.4	20(64.52)	Two	NR	1. A, 7(22.58) 2. B, 13(41.94) 3. C, 11(35.48)	NR	NR	1. From August 2009 to August 2010 2. Patients of MHE with Cirrhosis	1. Improvement in Psychometric tests 2. Improvement in MHE	"Prevalence of MHE is high in patients with cirrhosis of the liver. Rifaximin, LOLA, and probiotics are better than giving a placebo in patients with MHE"
		Rifamixin, 31(25)			43.9 ± 12.5	20(64.52)			1. A, 12(38.71) 2. B, 10(32.26) 3. C, 9(29.03)					
		Probiotics, 32(25.81)			33.87 ± 13.2	17(53.125)			1. A, 6(18.75) 2. B, 21(65.63) 3. C, 5(15.63)					
		Placebo, 30(24.19)			38 ± 11.8	20(66.67)			1. A, 10(33.33) 2. B, 8(26.67) 3. C, 12(40)					
34	Shehata, 2018 [47]	PEG, 50(50)	Egypt	RCT	54.5 ± 11.80	30(60)	NR	NR	NR	NR	NR	1. Signed the informed consent 2. Patients of MHE with Cirrhosis	1. Improvement in MHE 2. Adverse events	"Both lactulose and PEG were safe and effective in the treatment of HE. PEG significantly decreased the time needed for resolution of HE and significantly shortened the hospital stay"
		Lactulose, 50(50)			56.42±8.60	22(44)								
35	Sidhu, 2017 [48]	LOLA, 98(50.77)	India	RCT	49.6±10.5	89(90.8)	One	1. HCV, 19(19.4) 2. HBV, 13(13.3) 3. Alcohol, 16(16.3) 4. NASH, 5(5.1) 5. Other, 6(6.1)	1. A, 13(13.3) 2. B, 36(36.7) 3. C, 49(50)	20.6±8.3	NR	1. Patients with cirrhosis 2. Aged 18–75 years 3. Bout of OHE grade 2-4	1. Change in ammonia levels 2. Change in laboratory levels 3. MHE development 4. Safety and adverse events	"In patients with bouts of OHE, intravenous LOLA (as an add-on therapy to Lactulose and Ceftriaxone) significantly improves the grade of OHE over days 1-4, but not on day 5; decreases venous ammonia, decreases the time of recovery and shortens the length of hospital stay"
		Placebo, 95(49.23)			48.6±12.5	84(88.4)		1. HCV, 17(17.8) 2. HBV, 18(18.9) 3. Alcohol, 20(21.1) 4. NASH, 5(5.4) 5. Other, 4(4.4)	1. A, 14(14.7) 2. B, 41(43.2) 3. C, 43(43.3)	20.6±7.7				
36	Suzuki, 2017 [49]	Rifaximin, 84(49.12)	Japan	RCT	66 ± 6.167	43(51.2)	Three	1. HBV, 10(12) 2. HCV, 31(37.3) 3. AC, 18(21.7) 4. Others, 24(28.9)	1. A, 12(14.3) 2. B, 55(65.5) 3. C, 17(20.2)	NR	24.15± 8.81	1. LC or idiopathic PSS patients 2. With grade I or II HE	1. Change in ammonia levels 2. Change in laboratory levels 3. Adverse events	"The efficacy of rifaximin is good enough and well tolerated in Japanese patients with HE and hyperammonemia"
		Lactitol, 87(50.88)			64 ± 7.67	46(52.9)		1. HBV, 9(10.7) 2. HCV, 30(35.7) 3. AC, 24(28.6) 4. Others, 21(25)	1. A, 10(11.5) 2. B, 57(65.5) 3. C, 20(23)		24.4 ± 7.64			
37	Uchida, 2020 [50]	Rifaximin and Lactitol, 95(100)	Japan	Retrospective cohort study	68 ± 8.33	55(57.9)	36	1. HBV, 4(4.2) 2. HCV, 20(21.1) 3. Alcohol, 23(24.2) 4. NAFLD, 28(30.5) 5. AIH, 13(13.7) 6. PBC, 7(7.4)	1. A, 20(21.1) 2. B, 54(56.8) 3. C, 21(22.1)	NR	18 ± 11.96	1. Between January 2017 and February 2019 2. Informed consent was obtained from all the patients 3. Patients with decompensated cirrhosis	1. OHE occurrence rate 2. Change in Ammonia levels 3. Safety and adverse events	"Furosemide contributed to the deteriorated outcome of patients receiving rifaximin. Consequently, rifaximin should be given before increasing the furosemide dose, and the furosemide dose should not be increased during rifaximin treatment"
38	Varakanahalli, 2018 [51]	LOLA, 75(50)	India	RCT	44.39 ± 7		Six	1. Alcohol, 43(57.33) 2. HBV, 14(18.67) 3. HCV, 8(10.67) 4. AIH, 1(1.33) 5. NASH, 1(1.33) 6. Cryptogenic, 8(10.67)	9.04 (SD 1.528)	13.994 ± 2.745	96.73 ± 16.78	1. From March 2015 to October 2016 2. Patients with cirrhosis 3. Had no OHE	1. OHE Occurrence rate 2. Change in SIP	"LOLA is effective in the secondary prophylaxis of HE and is associated with significant improvements in psychometric hepatic encephalopathy score, ammonia level, critical flicker frequency scores, and health-related quality of life"
		Placebo, 75(50)			43.91 ± 7.1			1. Alcohol, 40(53.33) 2. HBV, 14(18.67) 3. HCV, 7(9.33) 4. AIH, 0 5. NASH, 1(1.33) 6. Cryptogenic, 13(17.33)	9.07 (SD 1.536)	14.136 ± 2.707	16.6 ± 2.785			
39	Wang, 2019 [52]	Lactulose, 67(68.37)	China	RCT	52.39 ± 10.81	60(89.6)	Two	1. HBV, 50(74.6) 2. HCV, 8(11.9) 3. Others, 9(13.5)	7.57 (SD 2.25)	NR	82.16 ± 47.40	1. Patients aged 18–70 years 2. Patients with cirrhosis and MHE	1. HRQOL Improvement 2. Safety and adverse events	"Treatment with lactulose significantly improves MHE recovery rate, and gut microbiota change in MHE patients can modulate the effectiveness of this therapy"
		Control, 31(31.63)			48.19 ± 10.73	31(100)		1. HBV, 18(58.1) 2. HCV, 4(12.9) 3. Others, 9(29)	7.03 (SD 2.14)		90.39 ± 47.33			
40	Yokoyama, 2022 [53]	Rifaximin, 12(100)	Japan	Retrospective cohort study	65.1 11.6	75(66.96)	36	1. Alcohol consumption(AC), 47(42) 2. Viral hepatitis (VH), 27(24.1) 3. NASH, 14(12.5) 4. AC and VH, 7(6.3) 5. AC and AIH, 1(0.9) 6. Others, 16(14.3)	9.54 (SD 2.1)	12.9 ± 4.43	20.94 ± 10.97	1. Adult patients with LC 2. Aged 20 years or older 3. With hyperammonemia who started oral RFX 4. Between January 2017 and December 2021	1. Change in Ammonia levels 2. Safety and adverse events	"RFX reduced blood ammonia concentration and improved hepatic spare ability and the quality of life of patients with long-term HE to up to 36 months. Our study revealed the effects of RFX against refractory ascites, suggesting that renin concentration may be a predictive marker for assessing ascites control"
41	Lv, 2020 [54]	Rifaximin, 50(66.67)	China	RCT	54.60 ± 9.05	42(84)	Six	HBV, HCV, Alcohol, HBV/alcohol, NASH, AIH and PBC	1. B, 16(64) 2. C, 9(36)	10 ± 1.75	NR	1. Cirrhotic patients with refractory ascites 2. Between November 2016 and May 2018 3. Age range 18-80 years	1. Change in Laboratory levels 2. Change in Clinical data 3. Adverse events	"Rifaximin mitigates ascites and improves survival of cirrhotic patients with refractory ascites. A possible mechanism is that rifaximin regulates the structure and function of intestinal bacteria, thus improving the systemic inflammatory state"
		Control, 25(33.33)			59.04 ± 10.01	18(72)			1. B, 21(42) 2. C, 29(58)	8 ± 1				
42	Ziada, 2013 [55]	Lactulose, 24(32)	Egypt	RCT	48.8 ± 8.2	18(75)	One	NR	1. A, 2(8.33) 2. B, 14(58.33) 3. C, 8(33.33)	NR	72.29 ± 24.50	1. Cirrhotic patients 2. From March 2010 to January 2012	1. OHE Development 2. Change in laboratory levels 3. Change in Ammonia levels	"Both probiotic and lactulose therapy can improve blood ammonia and psychometric tests in MHE and reduce the risk of developing overt encephalopathy. MRS showed more improvement in the levels of brain neurometabolites in the probiotic group"
		Probiotics, 26(34.67)			50.3 ± 7.8	19(73.1)			1. A, 3(11.54) 2. B, 14(53.85) 3. C, 9(34.62)		71.1 ± 19.67			
		Control, 25(33.33)			51.2 ± 7.5	18(72)			1. A, 3(12) 2. B, 13(52) 3. C, 9(36)		71.8 ± 15.01			
43	Egberts, 1984 [56]	BCAA, 11(50)	Germany	RCT	51 ± 6	7(63.64)	Three weeks	1. Alcohol, 9(81.82) 2. Hepatitis, 2(18.18)	NR	NR	NR	1. Patients with LC verified by biopsy specimen 2. All patients had clinical evidence of PH 3. None had clinical signs of PSE	1. Change in Ammonia levels 2. Psychometric test results	"Branched-chain amino acids appear to be effective in the short-term treatment of latent PSE and might be promising in long-term treatment"
		Placebo, 11(50)			52 ± 10	9(81.82)		1. Alcohol, 10(90.91) 2. Hepatitis, 1(9.09)						

Risk of Bias Assessment

**Figure 2:**
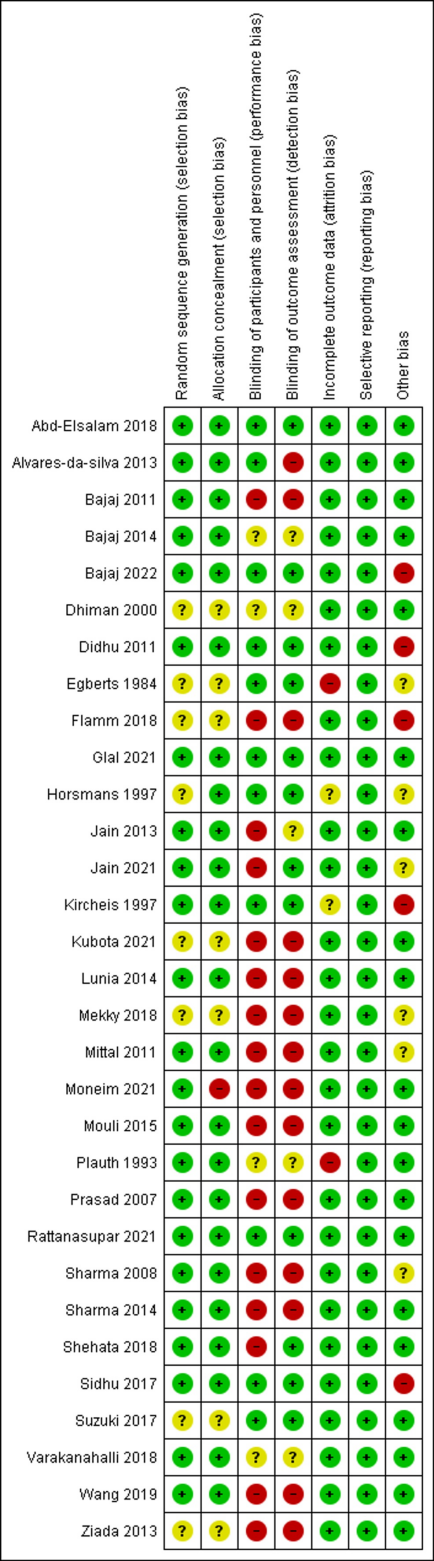
Risk of bias for randomized controlled trials References: [14,15,18,19,22-26,28,30-37,39,40,42-49,51,52,55,56].

RCTs assessed by the Cochrane ROB tool showed a high risk of bias, mostly due to the high risk of bias regarding blinding status. Figure 2 shows the summary of the risk of bias in RCTs.

Most of our observational studies assessed by the NIH tool showed fair quality; this could be attributed to insufficient sample size, not reporting the number of exposure assessments, and the patients were not blinded. The summary of cohort and case-control studies is shown in Table 2.

**Table 2:** NIH Quality Assessment tool NIH tool for cohort and cross-sectional studies. References: [16,17,20,21,27,29,38,41,50,53,54]

ID	NIH Quality Assessment Tool for Observational Cohort and Cross-Sectional Studies																Quality rating: good, fair or poor	
	1. Was the research question or objective in this paper clearly stated?	2. Were eligibility/selection criteria for the study population prespecified and clearly described?	3. Were the participants in the study representative of those who would be eligible for the test/service/intervention in the general or clinical population of interest?	4. Were all eligible participants that met the prespecified entry criteria enrolled?	5. Was the sample size sufficiently large to provide confidence in the findings?	6. For the analyses in this paper, were the exposure(s) of interest measured prior to the outcome(s) being measured?	7. Was the time frame sufficient so that one could reasonably expect to see an association between exposure and outcome if it existed?	8. For exposures that can vary in amount or level, did the study examine different levels of the exposure as related to the outcome (e.g., categories of exposure, or exposure measured as continuous variable)?	9. Were the exposure measures (independent variables) clearly defined, valid, reliable, and implemented consistently across all study participants?	10. Was the exposure(s) assessed more than once over time?	11. Were the outcome measures prespecified, clearly defined, valid, reliable, and assessed consistently across all study participants?	12. Were the people assessing the outcomes blinded to the participants' exposures/interventions?	13. Was the loss to follow-up after baseline 20% or less? Were those lost to follow-up accounted for in the analysis?	14. Were key potential confounding variables measured and adjusted statistically for their impact on the relationship between exposure(s) and outcome(s)?	Total scores			
	Yes / No / Not reported (NR) or cannot determine (CD) or not applicable (NA)	Yes / No / Not reported (NR) or cannot determine (CD) or not applicable (NA)	Yes / No / Not reported (NR) or cannot determine (CD) or not applicable (NA)	Yes / No / Not reported (NR) or cannot determine (CD) or not applicable (NA)	Yes / No / Not reported (NR) or cannot determine (CD) or not applicable (NA)	Yes / No / Not reported (NR) or cannot determine (CD) or not applicable (NA)	Yes / No / Not reported (NR) or cannot determine (CD) or not applicable (NA)	Yes / No / Not reported (NR) or cannot determine (CD) or not applicable (NA)	Yes / No / Not reported (NR) or cannot determine (CD) or not applicable (NA)	Yes / No / Not reported (NR) or cannot determine (CD) or not applicable (NA)	Yes / No / Not reported (NR) or cannot determine (CD) or not applicable (NA)	Yes / No / Not reported (NR) or cannot determine (CD) or not applicable (NA)	Yes / No / Not reported (NR) or cannot determine (CD) or not applicable (NA)	Yes / No / Not reported (NR) or cannot determine (CD) or not applicable (NA)				
Bai, 2019 [16]	Yes	Yes	Yes	NR	Yes	Yes	Yes	NR	NR	NR	Yes	NR	Yes	Yes	9	Fair		
Bai, 2019 (2) [17]	Yes	Yes	Yes	Yes	Yes	Yes	Yes	NR	NR	NR	Yes	NR	Yes	Yes	10	Fair		
Bajaj, 2019 [20]	Yes	Yes	Yes	Yes	Yes	Yes	Yes	NR	Yes	NR	Yes	NR	Yes	Yes	11	Good		
Chang, 2021 [21]	Yes	Yes	Yes	Yes	No	Yes	Yes	NR	Yes	NR	Yes	NR	Yes	Yes	10.5	Fair		
Fritz, 2022 [27]	Yes	Yes	Yes	Yes	NR	Yes	Yes	NR	NR	NR	Yes	NR	Yes	Yes	10	Fair		
Hiramine, 2021 [29]	Yes	Yes	Yes	Yes	No	Yes	Yes	NR	Yes	NR	Yes	NR	Yes	Yes	10.5	Fair		
Miwa, 2022 [38]	Yes	Yes	NR	NR	No	Yes	Yes	NR	NR	NR	Yes	NR	Yes	Yes	7.5	Fair		
Okada, 2020 [41]	Yes	Yes	Yes	NR	Yes	Yes	Yes	NR	Yes	NR	Yes	NR	Yes	Yes	10	Fair		
Uchida, 2020 [50]	Yes	Yes	Yes	NR	NR	Yes	Yes	NR	Yes	NR	Yes	NR	Yes	Yes	10	Fair		
Yokoyama, 2022 [53]	Yes	Yes	Yes	Yes	NR	Yes	Yes	NR	NR	NR	Yes	NR	Yes	Yes	9	Fair		
Lv, 2020 [54]	Yes	Yes	Yes	Yes	NR	Yes	Yes	NR	Yes	NR	Yes	NR	Yes	Yes	10	Fair		

Outcomes

Development of Overt HE

**Figure 3:**
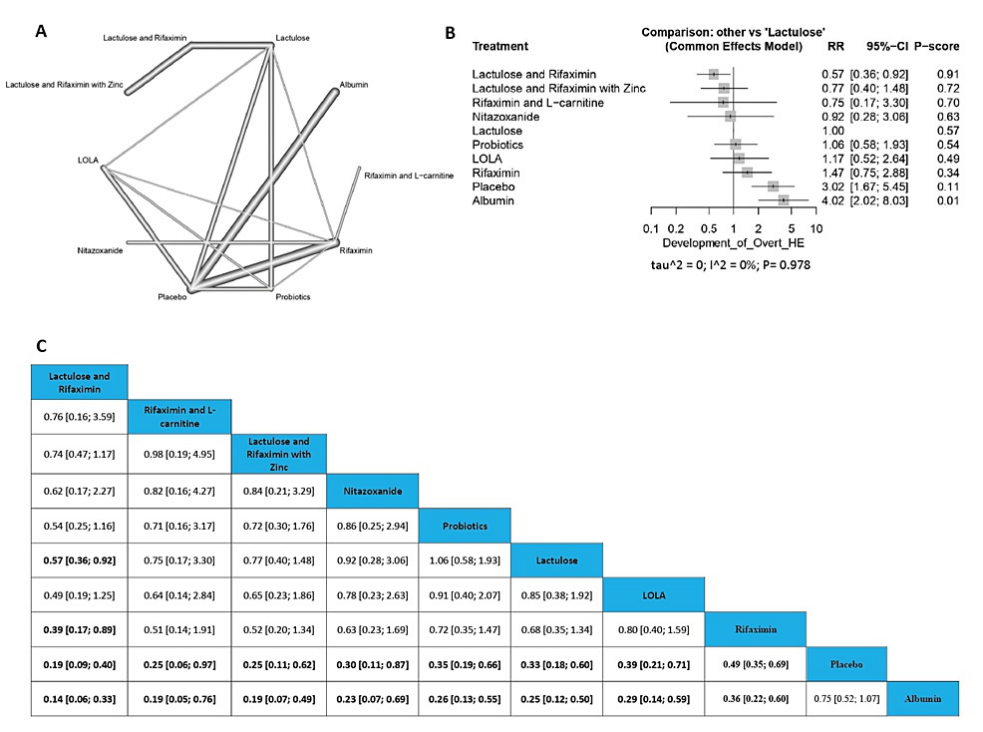
Development of overt HE

The results showed a significant reduction in the incidence of development of overt HE between (Lactulose and Rifaximin) compared with Lactulose (RR=0.57, 95% CI [0.36; -0.92]), in addition to a significant variation in cases of (Lactulose and Rifaximin) compared with Rifaximin (RR=0.39, 95% CI [0.17;0.89]) with another significantly lower incidence of development of overt HE in (Lactulose and Rifaximin) compared with placebo (RR=0.19, 95% CI [0.09; 0.40]). Most of the arms showed a significant reduction in the incidence of development of overt HE compared with albumin and placebo. The order of drugs in terms of incidence of development of overt HE according to P-score is (Lactulose and Rifaximin), (Rifaximin and L-carnitine), (Lactulose and Rifaximin with zinc), Nitazoxanide, Probiotics, Lactulose, L-ornithine L-aspartate, Rifaximin, placebo, and Albumin (Figure 3).

Reduction in Ammonia

**Figure 4:**
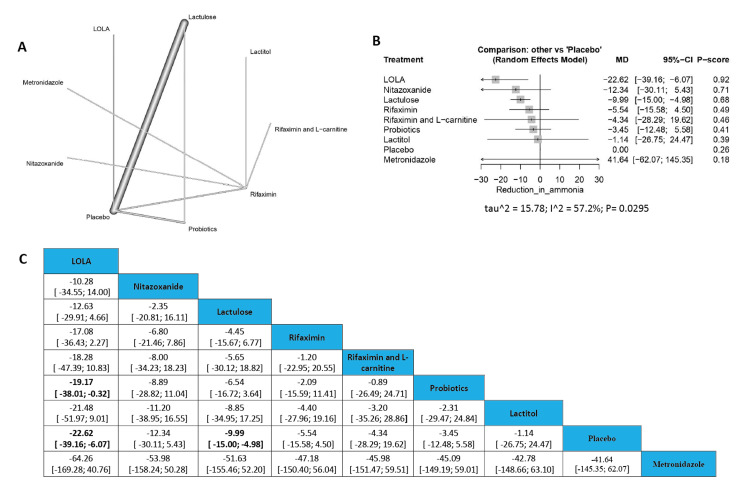
Reduction in ammonia

The results showed a significant reduction in ammonia between L-ornithine L-aspartate and probiotics (MD=-19.17, 95% CI [-38.01; -0.32]), in addition to a significant variation in cases of L-ornithine L-aspartate compared with placebo (MD=-22.62, 95% CI [-39.16; -6.07]) with another significant reduction in ammonia in the lactulose compared with placebo (MD=-9.99, 95% CI [-15.00; -4.98]). The order of drugs in terms of incidence of reduction in ammonia according to P-score is L-ornithine L-aspartate, Nitazoxanide, Lactulose, Rifaximin, (Rifaximin and L-carnitine), Probiotics, Lactitol, Placebo, and Metronidazole (Figure 4).

Reversal of Minimal HE

**Figure 5:**
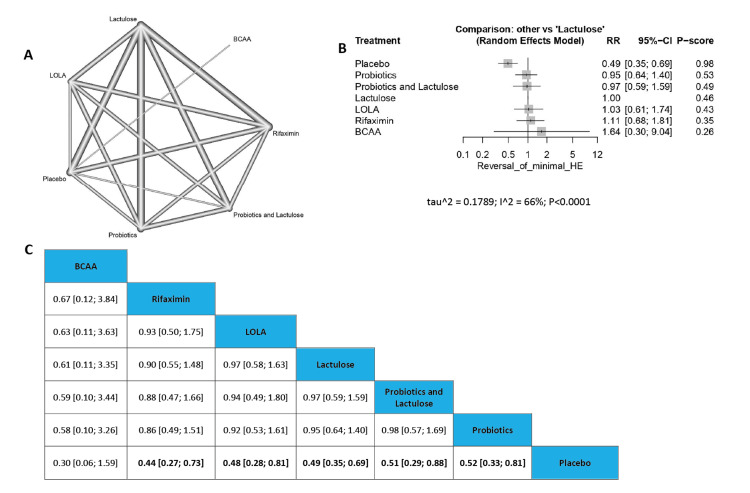
Reversal of minimal HE

In the outcome of reversal of minimal HE all the significant results were with placebo which is expected, so we find the only non-significant results compared to Placebo were with branched-chain amino acids (RR=0.30, 95% CI [0.06; 1.59]), on the other hand, Placebo showed a significant result compared with Rifaximin (RR=0.44, 95% CI [0.27; 0.73]), and with Lactulose (RR=0.49, 95% CI [0.35; 0.69]) respectively. The order of drugs in terms of reversal of minimal HE according to P-score from low to high is Placebo, Probiotics, Probiotics and Lactulose, Lactulose, L-ornithine L-aspartate, Rifaximin, and branched-chain amino acids (Figure 5).

Adverse Events

**Figure 6:**
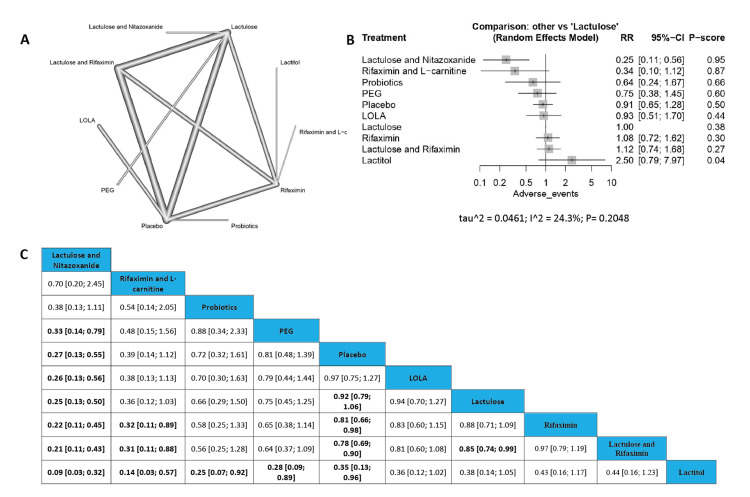
Adverse events

The results showed a significant variation between (Lactulose and Nitazoxanide), and Lactitol (RR=0.09, 95% CI [0.03; 0.32]), in addition to a significant variation in cases of [Lactulose and Nitazoxanide] compared with placebo [RR=0.27, 95% CI [0.13; 0.55]) with another significantly lower incidence of adverse events in (Rifaximin and L-carnitine) compared with Rifaximin only (RR=0.32, 95% CI [0.11; 0.89]), with another significantly lower incidence of adverse events in (Lactulose and Nitazoxanide) compared with (Lactulose and Rifaximin) (RR=0.31, 95% CI [0.11; 0.88]). Moreover, many significant results appeared to be shown in the comparison between (Lactulose and Nitazoxanide), and (Rifaximin and L-carnitine) compared to the other arms. The ranking of drugs in terms of incidence of adverse events according to P-score is (Lactulose and Nitazoxanide) followed by (Rifaximin and L-carnitine), Probiotics, PEG, Placebo, L-ornithine L-aspartate, Lactulose, Rifaximin, (Lactulose and Rifaximin), and Lactitol (Figure 6).

All Causes of Mortality

**Figure 7:**
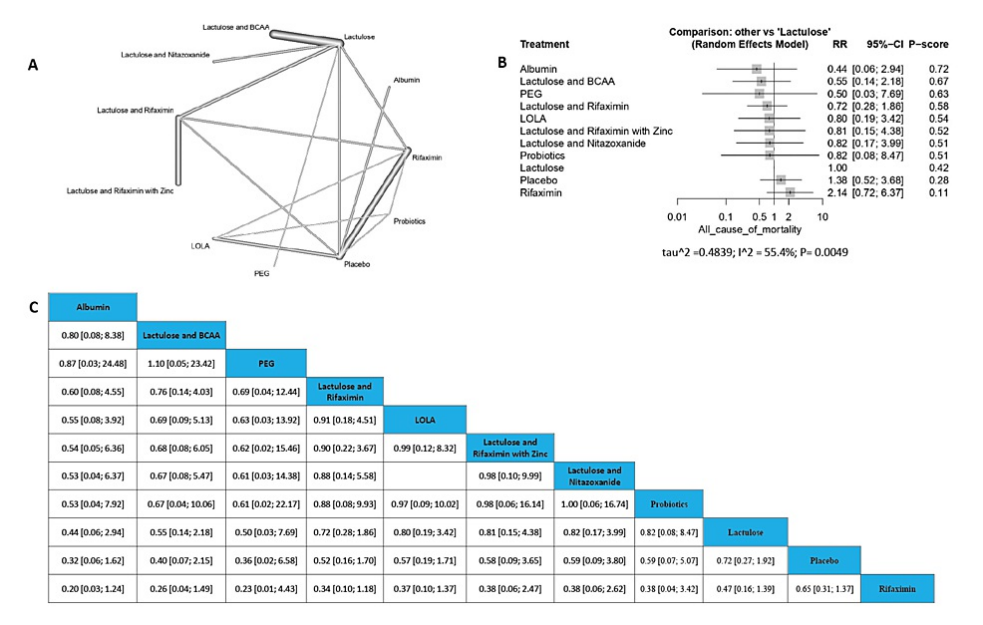
All causes of mortality

The results showed no significant decrease in all causes of death in the comparison among all the tested medications compared with the control. The order of drugs in terms of all causes of mortality incidence according to P-score is Albumin followed by (Lactulose and BCAA), PEG, (Lactulose and Rifaximin), L-ornithine L-aspartate, (Lactulose and Rifaximin with zinc), (Lactulose and Nitazoxanide), Probiotics, Lactulose, Placebo, and finally Rifaximin (Figure 7).

Change in Health-Related Quality of Life (HRQOL)

**Figure 8:**
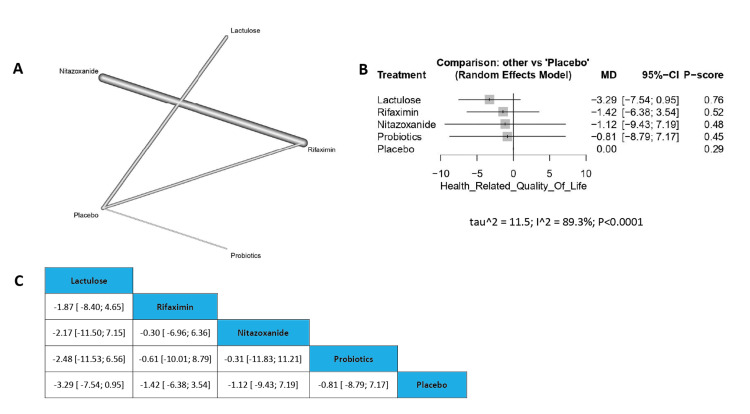
Change in HRQOL HRQOL: Health-related quality of life

No available treatment was found significant in the improvement of health-related quality of life, as lactulose showed no significant result compared to placebo (MD=-3.29, 95% CI [-7.54; 0.95]), and the order of drugs in terms of change in HRQOL according to P-score is Lactulose, Rifaximin, Nitazoxanide, Probiotics, and placebo (Figure 8).

Discussion

In our network meta-analysis, 32 RCTs and 11 cohorts were included with a total population of 19622. Our results showed that lactulose and rifaximin were significant compared to most of the arms in the outcome of the development of overt HE, in addition to the reversal of minimal HE that showed significance in the placebo group. Moreover, the reduction in ammonia showed significance in the L-ornithine L-aspartate drug compared to other groups. No significant results were found in HRQOL as all arms had the same effect on the quality of life. Hepatic encephalopathy was found to impair daily function and driving skills due to a decrease in cognition [9, 57]. HE increases the burden on medical care providers, especially with the poor prognosis for HE in all its forms [58]. Some papers found that a combination of Rifaximin and Lactulose would benefit in decreasing mortality [59], although our study showed no significance among the available treatment options as they all would have decreased mortality, no results are significant. In our study results, Lactulose was a common factor in most of our significant results and showed the highest rate of improvement in the quality of life [60], which matched previous studies in addition to the benefit of cost reduction due to the cheap cost of lactulose usage compared to other drugs [61].

L-ornithine L-aspartate showed the highest reduction in serum ammonia in our results and the results were significant compared to the other arms, the serum ammonia is one of the core factors for hepatic encephalopathy pathogenesis. A previous study proved the effectiveness of L-ornithine L-aspartate in decreasing the ammonia levels in HE [62]. Although the combination between Lactulose and nitazoxanide is showing promising results in adverse event outcomes, more studies investigating this arm are still required with higher populations [62, 63]. All our included drugs were well tolerable when it comes to adverse events even the lactulose except for nausea, bloating, and diarrhea but all these side effects could be overcome with dose adjustments or the addition of nitazoxanide [63]. This analysis allowed us to compare available treatment options for achieving clinically relevant endpoints and provided a ranking of each treatment's efficacy. This method was the best alternative to a direct comparison study for comparing interventions for HE.

The strength points of this study are that it contains a large number of RCTs in addition to cohort studies. Our study has a high total population as 19622 patients were included, which would provide stronger evidence as long as the RCTs would provide more solid data with low bias, as RCTs are the gold standard for strong evidence. Our study also contained variable arms with different comparisons between these arms as we almost included every available way of treatment for hepatic encephalopathy in the market, every single part of our meta-analysis was reviewed at least twice, moreover all PRISMA recommendations were followed while making this network meta-analysis. Our study also had some limitations as not all inclusion criteria for patients with hepatic encephalopathy were the same. The duration of the follow-up period wasn’t equal in all included studies. In addition, some of the doses were different and we made no subgroups for the different doses.

## Conclusions

We provided an extensive body of evidence for the management of subclinical HE in patients without a history of overt HE which is compiled in this network meta-analysis (NMA). We can show evidence of the development of overt HE (Lactulose and Rifaximin followed by Rifaximin and L-carnitine, and followed by Lactulose and Rifaximin with zinc). According to the reduction of ammonia, LOLA was ranked first, followed by Nitazoxanide, and finally Lactulose. The order of incidence of adverse events is lowest in (Lactulose and Nitazoxanide) followed by (Rifaximin and L-carnitine) and finally Probiotics. On the other hand, no valuable results were obtained from all causes of mortality outcomes and changes in the quality of life. Further studies in the future are required to investigate our interventions with direct comparisons among all of our interventions.
